# Antiviral Activity of the Rhamnolipids Mixture from the Antarctic Bacterium *Pseudomonas gessardii* M15 against Herpes Simplex Viruses and Coronaviruses

**DOI:** 10.3390/pharmaceutics13122121

**Published:** 2021-12-08

**Authors:** Rosa Giugliano, Carmine Buonocore, Carla Zannella, Annalisa Chianese, Fortunato Palma Esposito, Pietro Tedesco, Anna De Filippis, Massimiliano Galdiero, Gianluigi Franci, Donatella de Pascale

**Affiliations:** 1 Department of Experimental Medicine, University of Campania “Luigi Vanvitelli”, 80138 Naples, Italy; rosa.giugliano@unicampania.it (R.G.); carla.zannella@unicampania.it (C.Z.); annalisa.chianese@unicampania.it (A.C.); anna.defilippis@unicampania.it (A.D.F.); massimiliano.galdiero@unicampania.it (M.G.); 2 Department of Marine Biotechnology, Stazione Zoologica Anton Dohrn, Villa Comunale, 80121 Naples, Italy; carmine.buonocore@szn.it (C.B.); fortunato.palmaesposito@szn.it (F.P.E.); pietro.tedesco@szn.it (P.T.); 3 Institute of Biochemistry and Cell Biology, National Research Council, 80131 Naples, Italy; 4 Department of Medicine, Surgery and Dentistry, “Scuola Medica Salernitana”, University of Salerno, 84081 Baronissi, Italy

**Keywords:** microbial biosurfactants, rhamnolipids, antiviral, SARS-CoV-2, enveloped virus, coronavirus, herpes, Antarctic bacteria, TEM

## Abstract

Emerging and re-emerging viruses represent a serious threat to human health at a global level. In particular, enveloped viruses are one of the main causes of viral outbreaks, as recently demonstrated by SARS-CoV-2. An effective strategy to counteract these viruses could be to target the envelope by using surface-active compounds. Rhamnolipids (RLs) are microbial biosurfactants displaying a wide range of bioactivities, such as antibacterial, antifungal and antibiofilm, among others. Being of microbial origin, they are environmentally-friendly, biodegradable, and less toxic than synthetic surfactants. In this work, we explored the antiviral activity of the rhamnolipids mixture (M15RL) produced by the Antarctic bacteria *Pseudomonas gessardii* M15 against viruses belonging to *Coronaviridae* and *Herpesviridae* families. In addition, we investigated the rhamnolipids’ mode of action and the possibility of inactivating viruses on treated surfaces. Our results show complete inactivation of HSV-1 and HSV-2 by M15RLs at 6 µg/mL, and of HCoV-229E and SARS-CoV-2 at 25 and 50 µg/mL, respectively. Concerning activity against HCoV-OC43, 80% inhibition of cytopathic effect was recorded, while no activity against naked Poliovirus Type 1 (PV-1) was detectable, suggesting that the antiviral action is mainly directed towards the envelope. In conclusion, we report a significant activity of M15RL against enveloped viruses and demonstrated for the first time the antiviral effect of rhamnolipids against SARS-CoV-2.

## 1. Introduction

Viruses can be structurally divided in two groups according to the presence or not of lipids as integral components of their superficial structure [1]. Viruses acquire these lipids during viral assembly and exit from the infected cell, generally through a buddings mechanism, which leads to the formation of a membrane that envelops the capsid containing the viral genome [2]. This lipidic layer, called the viral envelope, is shared by many different families of viruses, such as retrovirus, herpesvirus, coronavirus, orthomyxovirus, rhabdovirus, filovirus, and paramyxovirus, despite the great diversity in their structure, replication strategy, pathogenesis, host-range, and diseases that they cause [3]. This structure is a key determinant of viral entry into the host cell, a paramount step to start any infectious cycle, and therefore it is an important target for antiviral drugs and disinfectants [4,5].

Herpes simplex virus type 1 (HSV-1) and 2 (HSV-2) are enveloped viruses and are the etiological agents of labial and genital herpes, respectively. They present a double-stranded DNA genome and belong to the *Herpesviridae* family [6]. Moreover, HSV-1, HSV-2, and Varicella-Zoster virus (Human alphaherpesvirus 3) are grouped in the subfamily *Alphaherpesvirinae* for their relatively fast life cycle [7]. Even if most of the times they do not cause any symptomatology, both HSV-1 and HSV-2 are lifelong, recurrent pathogens, and can cause blistering or be fatal in immunocompromised people [8]. The resurgence of these viruses is due to their ability to remain in a latent state, within sensory neurons, and eventually being reactivated to give rise to a novel infectious cycle [9]. The World Health Organization (WHO) estimates that more than 60% of people worldwide are infected with HSV-1. At the same time, genital infection with HSV-2 is associated with an increased probability of contracting human immunodeficiency virus (HIV) infections [10,11]. 

Another enveloped virus that reached the pandemic level is SARS-CoV-2, the etiological agent of COVID-19 that caused more than 200 million cases and almost 5 million deaths in the last two years [12].

SARS-CoV-2 is a member of the Human coronaviruses (HCoV) group, that are positive-sense, single-stranded RNA viruses belonging to the *Coronaviridae* family [13]. Since their first identification in 1965, seven members belonging to *alpha*- and *betacoronavirus* genera are known to infect humans: human coronavirus strain 229E (HCoV-229E) and human coronavirus strain NL63 (HCoV-NL63), belonging to *alphacoronaviruses*, and human coronavirus strain OC43 (HCoV-OC43), human coronavirus strain HKU1 (HCoV-HKU1), Severe Acute Respiratory Syndrome coronavirus (SARS-CoV), Middle East Respiratory Syndrome coronavirus (MERS-CoV), and Severe Acute Respiratory Syndrome coronavirus 2 (SARS-CoV-2), belonging to the *betacoronaviruses* [14]. HCoV-229E and HCoV-OC43 are models of the two main genera of coronaviruses (alpha and beta, respectively) that can cause common colds and are globally distributed [13,15]. 

Due to their contagiousness and consequences for human health, there is a high interest in suppressing their diffusion worldwide. A potential answer can be represented by microbial biosurfactants. Biosurfactants are amphiphilic compounds capable of lowering surface tension between liquids, and possess several biological activities [16,17,18,19,20,21]. Furthermore, the interest in biosurfactant-based products and their applications is growing in recent years because they present lower cytotoxicity and higher biodegradability when compared to chemical surfactants [22,23].

RLs are the most well-known and characterized anionic biosurfactants, generally produced by some species of *Pseudomonas* and *Burkholderia,* and consist of one or two rhamnose molecules and up to three hydroxyl fatty acid molecules containing 8–14 carbon atoms [24,25]. RLs possess a wide range of industrial applications and are already commercially used as additives in skincare products and cosmetics (i.e., TeeGenes and Evonik) [26,27,28,29,30]. RLs also showed a broad-spectrum of bioactivities, such as antibacterial, antifungal, antitumoral, antibiofilm, antioxidant, and can also be applied for drug delivery [31,32,33,34,35,36]. Moreover, RLs showed antiviral activity against HSV-1, HSV-2, and bovine coronavirus, interacting with lipid membranes of viruses, inducing changes in viral membrane glycoproteins [37,38]. However, their mechanism of action has not been completely elucidated [22,39]. 

In our previous study, we characterized a new mixture of RLs endowed with antibacterial activity produced by the Antarctic strain *Pseudomonas gessardii* M15 by LC-MS/MS [40]. In this work, we investigate the ability of this mixture (M15RL) to inactivate enveloped viruses belonging to the *Herpesviridae* and *Coronaviridae* families, and in particular SARS-CoV-2, with a focus on their mode of action. To the best of our knowledge, they have never been tested against human coronaviruses, in particular against SARS-CoV-2. Furthermore, we explored and assessed the ability of M15RL to inactivate enveloped viruses on treated surfaces.

## 2. Materials and Methods

### 2.1. Rhamnolipids

Rhamnolipids produced by *P. gessardii* M15 were described and characterized in a previous work [40]. For their production, *P. gessardii* M15 was cultivated in a minimal medium supplemented with glycerol for 5 days. Then the culture was centrifuged, the supernatant was collected and extracted by using ethyl acetate. The obtained extract was fractionated by solid-phase fractionation at increasing methanol concentrations in water (65%, 80%, and 100%). The fraction eluted at 80% of methanol was named M15RL and utilized in all the tests described in this work. M15RL contains 17 mono-rhamnolipids, six of which (ca. 20% of the mixture, Table S1) were novel and were described for the first time in the previous work [40].

### 2.2. Cell Line and Viruses

Vero cells CCL-81, derived from the kidney’s epithelium of an African green monkey (*Cercopithecus aethiops*), were purchased from American Type Culture Collection (ATCC, Manassas, VA, USA), while the HaCaT cell line (human keratinocytes cells) was obtained from the CLS–Cell Lines Service (Eppelheim, Germany). Both cell lines were maintained in Dulbecco’s Modified Eagle Medium (DMEM) (Microgem, Naples, Italy) in a humidified atmosphere with 5% CO_2_ at 37 °C. DMEM composition: 4.5 g/L glucose, 2 mM l-glutamine, 100 IU/mL penicillin-streptomycin solution, and supplemented with 10% Fetal Bovine Serum (FBS) (Microgem, Naples, Italy).

All viruses were propagated on a Vero cell monolayer and included: HCoV-229E (ATCC VR-740), HCoV-OC43 (ATCC VR-1558), HSV-1 strain SC16, fluorescent HSV-1 (GFP-HSV-1), HSV-2 strain 333, Poliovirus Type 1 (PV-1) strain Chat (ATCC VR-1562), and Severe Acute Respiratory Syndrome (SARS-CoV-2) strain VR PV10734 clinical isolate, kindly provided by Lazzaro Spallanzani Hospital (Rome, Italy) (Table 1).

Viruses were maintained in the laboratory and stored at −80 °C until further use. All experimental work involving viruses was performed in an appropriate biosafety level containment laboratory.

### 2.3. Cytotoxicity Assay 

Vero and HaCaT cells (2 × 10^4^ cell/well) were seeded in 96-well plates and then incubated at 37 °C in a humidified atmosphere. After 24 h, cell monolayers were incubated in the absence or presence of serial dilutions of M15RL. The cytotoxicity was evaluated by methylthiazolyldiphenyl-tetrazolium bromide (MTT) assay (Sigma-Aldrich, St. Louis, MO, USA). Cell viability was determined 24 h after the treatment. MTT water solution (0.5 mg/mL) was added to the cells for 3 h, and 100 µL of dimethyl sulfoxide (DMSO) was added to each well to dissolve the formazan crystal. The cells viability was evaluated by recording the absorbance (Abs) at the wavelength of 570 nm with a TECAN M-200 reader (Tecan, Männedorf, Switzerland). The percentage of cell viability was calculated according to the following formula:

(1) $$\mathrm{Cell}\;\mathrm{viability}\;\left(\%\right)=\frac{\mathrm{Absorbance}\;\mathrm{of}\;\mathrm{treated}\;\mathrm{samples}}{\mathrm{Absorbance}\;\mathrm{of}\;\mathrm{not}\;\mathrm{treated}\;\mathrm{cells}}\times 100$$

### 2.4. Antiviral Assays

To evaluate the antiviral activity of M15RL, four different tests were performed. In all the following treatments, Vero cells were plated in 12-wells at an initial density of 2 × 10^5^ cells/well and incubated overnight at 37 °C. Then, cells were treated with M15RL at concentrations ranging from 50 to 0.7 µg/mL, and the virus was added (according to the test) at 0.01 or 0.1 multiplicity of infection (MOI). M15RL’s activity against HSV-1, HSV-2, HCoV-229E, PV-1, and SARS-CoV-2 was determined by plaque reduction assays in infected cell monolayers [41]. Antiviral activity against HCoV-OC43 was based on the inhibition of the cytopathogenicity induced by the virus in Vero cells. Pleconaril at 1 µM was used as a positive control against PV-1. As a positive control (CTRL+) for the other viruses, we utilized an internal standard named Greco extract at 50 µg/mL. This extract was obtained from the grape cane of *Greco* cultivar of *Vitis vinifera* and characterized in a previous work [42,43]. The negative control was represented by untreated infected cells. In treated cells, the antiviral activity was calculated as the percentage of plaque reduction compared to the untreated infected cells (CTRL–), in accordance with the following formula:

(2) $$\mathrm{Antiviral}\;\mathrm{activity}\;\left(\%\right)=\left(1-\frac{\mathrm{number}\;\mathrm{of}\;\mathrm{plaques}\;\mathrm{in}\;\mathrm{treated}\;\mathrm{cells}}{\mathrm{number}\;\mathrm{of}\;\mathrm{plaques}\;\mathrm{in}\;\mathrm{untreated}\;\mathrm{cells}}\right)\times 100$$

#### 2.4.1. Co-treatment Assay

The M15RL mixture was dissolved in DMEM at the selected concentrations, in the presence of viruses at 10^3^ Plaque Forming Units (PFU), and was immediately dispensed on the cells in free FBS medium for 1 h at 37 °C. Subsequently, the supernatant was removed, and the cell monolayer was washed with Phosphate Buffer Saline (PBS) (Microgem, Naples, Italy) and incubated in a fresh culture medium supplemented with DMEM plus 5% carboxymethylcellulose (CMC) (Sigma-Aldrich, St. Louis, MO, USA) for 24 h or 48 h (based on the virus). After the incubation, the cells were washed twice with PBS, fixed with 4% formaldehyde and stained with 0.5% crystal violet [43].

#### 2.4.2. Virus Pre-treatment Assay

M15RL was dissolved in FBS-free medium at different concentrations and pre-incubated in the presence of viral suspension at 10^4^ PFU for 1 h at 37 °C. After the incubation, each mixture was diluted in FBS-free DMEM and used to infect Vero cells for 1 h. Following a 1 h of viral absorption, the cell monolayer was washed with PBS, and then a fresh culture medium supplemented with 5% CMC was added. The plates were incubated 24 h for HCoV-229E and 48 h for the other viruses. After the incubation, the cells infected were processed, and the percentage of inhibition was calculated as described above.

Differently, for HCoV-OC43, the inhibition of cytopathic effect on Vero cells in the presence and absence of M15RL was evaluated. The cell viability was determined through the colorimetric MTT assay and 2 × 10^4^ cells/well were plated in a 96-well plate and incubated for 24 h at 37 °C in a humidified CO_2_ (5%) atmosphere. Cell monolayers were then infected with 100 μL of a virus dilution in DMEM. At the same time, 100 μL of the medium, without or with serial dilutions of M15RL, were added. After 72 h of incubation at 37 °C, cell viability was determined by adding 100 µL MTT solution and incubating for 3 h. Then, 100 µL of DMSO was added to each well to solubilize the formazan crystals. Finally, the absorbance was read at 570 nm with a microplate reader.

#### 2.4.3. Post-treatment Assay

Vero cells were first infected (MOI 0.01) for 1 h at 37 °C, then non penetrated virions were removed by washing gently with PBS. Finally, the cells were treated with rhamnolipids and incubated at 37 °C for 1 h. At the end of the treatment, the cell monolayer was washed with PBS 1X and incubated for 48 h in fresh medium.

#### 2.4.4. Cell Pre-treatment

The cells were pre-treated for 1 h with different concentrations of M15RL to verify the action on the target Vero cells. After, the compound was removed, and the cell monolayers were infected with the virus for 1 h at 37 °C. After viral adsorption, the supernatant was removed, and the cells were overlaid with DMEM supplemented with 5% CMC. After incubation, the plate was processed and analyzed as described above.

#### 2.4.5. Viral Attachment and Entry/Fusion Assay

Two separate tests were carried out to verify direct action on viral particles (i) attachment assay and (ii) entry/fusion assay. The tests were conducted as described by Tai et al. [44], with minor modifications, as follows:

- 2 × 10^5^ cells/well were seeded in a 12-well plate and incubated at 37 °C overnight. The plate was pre-chilled to 4 °C for 30 min before testing. The cells were co-treated with virus and substance in the 1:1 ratio, and the plate was incubated at 4 °C for 1 h. Then, the monolayer was washed twice with PBS, rinsed with fresh medium, and the plate was incubated at 37 °C for 48 h.
- Vero cells (2 × 10^5^ cells/well) were plated in a 12-well plate and incubated for 24 h in a humidified atmosphere with 5% CO_2_ at 37 °C. Before testing, the cell monolayer was cooled to 4 °C. The cells were infected with the virus (10^3^ PFU/mL) and were incubated at 4 °C for 1 h. Successively, the supernatant was removed, and the cell monolayer gently washed to remove the excess virus. Then, M15RL was added to each well, and the plate was incubated at 37 °C for 1 h. Finally, the supernatant was removed, rinsed with fresh medium, and incubated for 48 h.

### 2.5. Real-Time PCR

The antiviral potential of M15RL has also been verified through molecular tests (Real-time PCR). Vero cells (2 × 10^5^ cells/well) were plated in 12-well plates and infected with HSV-1 and SARS-CoV-2 as described in Section 2.4.2. After the incubation, the cells were collected and the total RNA extracted with TRIzol^®^ (Thermo Fisher, Waltham, MA, USA), and then quantified at the nanodrop (NanoDrop 2000, Thermo Fisher Scientific, Waltham, MA, USA). Finally, RNA was retrotranscribed to cDNA by SensiFAST™ cDNA Synthesis Kit (Meridian Bioscience, Washington, DC, USA). Real-Time PCR (RT-PCR) was run in triplicate using a CFX Thermal Cycler (Bio-Rad, Hercules, CA, USA). Then, 2 µL of cDNA were amplified in a final volume of 20 µL reactions using 10 µL of 2x SensiFAST™ SYBR^®^ No-ROX Mix (Meridian Bioscience, Washington, DC, USA) and 0.4 µM of each primer. The relative target threshold cycle (Ct) values of UL52 and UL27 (for HSV-1), S protein (for SARS-CoV-2) were normalized to Glyceraldehyde 3-phosphate dehydrogenase (GAPDH), as the housekeeping gene. Relative expression of the gene of interest (GOI) was calculated with the ∆C_T_ method, using GAPDH as the reference gene, in accordance with the following formula:

(3) $$\mathrm{Ratio}\;\left(\mathrm{reference}/\mathrm{target}\right)=2^{C_{T}\left(\mathrm{reference}\right)-C_{T}\left(\mathrm{target}\right)}$$

RT-PCRs were run as follows: 95 °C for 2 min (for Polymerase activation) and 95 °C for 5 s (Denaturation), plus 60 °C for 10 s (Annealing) and 72 °C for 20 s (Extension), for 40 cycles. Primers were purchased by Eurofins (Eurofins Biolab, Milan, Italy), and their sequences are designated in the following table (Table 2):

### 2.6. Microscopy

The antiviral effect of M15RL was also investigated with fluorescent microscopy on GFP-HSV-1, and electron microscopy, on HSV-1 and SARS-CoV-2. 

#### 2.6.1. Fluorescent Microscopy

GFP-HSV-1 was pre-treated as described in Section 2.4.2. Briefly, 10^4^ PFU of viral suspension was treated with different concentrations of M15RL for 1 h at 37 °C and then titrated on cells for 1 h. Then cells were washed, fresh medium supplied with 5% CMC was added and incubated for 48 h. Differently from the other viruses, plates infected with GFP-HSV-1 were not further processed. Images were acquired through the Nikon ECLIPSE Ti2-U (Nikon Europe B.V., Amsterdam, Netherlands) inverted fluorescence microscope with beam settings for FITC and BF.

#### 2.6.2. Transmission Electron Microscopy

Six 75 cm^2^ flasks, seeded with 90% Vero confluent cells, were infected with 0.01 MOI HSV-1 and SARS-CoV-2. The supernatant was collected when 80–90% cytopathic effect occurred and centrifuged at 300× *g* for 10 min to remove cellular debris, and was then subjected to an additional centrifuge at 2000× *g* for 10 min. In addition, three ultracentrifugation cycles were carried out to collect viral particles, the first at 10,000× *g* for 30 min, then at 100,000× *g* for 70 min. Ultracentrifugation steps were performed through an Optima XPN Beckman equipped with SW 32 Ti Swinging-Bucket Rotor Package (Beckman Coulter, Milan, Italy). Viral load was assessed by plaque assay on a Vero cell monolayer (Section 2.4.1) and the PFU was counted. To visualize the effect of M15RL on viral particles, a drop of 5 µL of virus (10^8^ PFU) was incubated with 5 µL of M15RL (50 µg/mL) 1 h at 37 °C. Controls were incubated only with DMEM medium. Then, 5 µL of the suspension was transferred to formvar/carbon-coated copper grids, 200 mesh (Sigma-Aldrich, St. Louis, MO, USA). Finally, the samples were stained with 2% phosphotungstic acid, pH 6.5, for 30 s to enhance the contrast. Images were obtained with a ZEISS LEO 912 AB (ZEISS, Oberkochen, Germany) Transmission Electron Microscope (TEM).

### 2.7. Coating of Plastic Surface with M15RL Mixture

For this task, a stock solution of M15RL dissolved in methanol (MeOH) at 10 mg/mL was prepared. Then, the bottom surface of a 12-well plate, ~4 cm^2^, was coated with 10 µL of the suspension at concentrations ranging from 50 to 0.7 µg/mL. The MeOH was evaporated for 20 min under the microbiological hood in sterile conditions. Then, 10 µL of viruses containing 10^4^ PFU were added directly to the bottom of the well for 5 min. The direct contact between the plastic surface and the virus was interrupted by adding 1 mL of PBS for 1 h at 37 °C. Finally, 100 µL of the PBS suspension from each well was titrated by standard plaque reduction assay. As controls, the wells were treated only with MeOH to check if the vehicle influenced the virus, while untreated wells were tested to verify the integrity of the virus on the surface.

### 2.8. Statistical Analysis

All tests were performed in triplicate and expressed as mean ± Standard Deviation (SD), One-way ANOVA followed by the Dunnett’s multiple comparisons test, Two-way ANOVA followed by Sink’s multiple comparisons test, and graphs were generated using GraphPad Prism ver. 8.2.1 for macOS (GraphPad Software, San Diego, CA, USA, www.graphpad.com).

## 3. Results

### 3.1. Antiviral Activity

**Figure 1:**
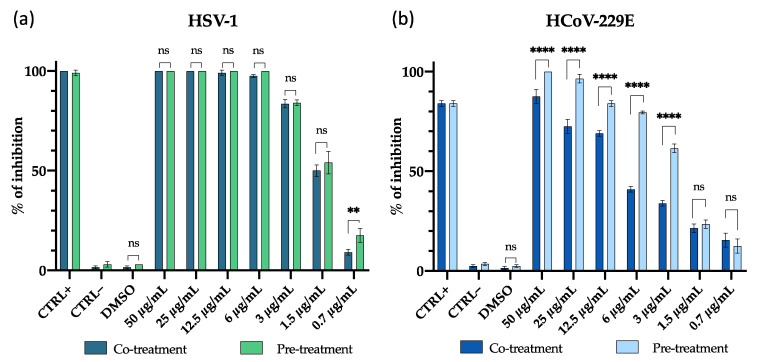
Antiviral activity of M15RL, presented as percentage of inhibition, against (**a**) HSV-1 and (**b**) HCoV-229E in the co-treatment assay (cells treated with virus and M15RL at the same time) and pre-treatment assay (virus treated with M15RL for 1 h and then titrated on cells). The inhibition percentage was calculated by comparing the number of plaques found in the presence of M15RL with respect to the untreated virus (CTRL–) (Formula (2)). The Greco extract [42] at 50 µg/mL was used as a positive control (CTRL+). Data are means of three independent experiments. Statistical analyses were determined by two-way ANOVA with Sidak’s test for multiple comparisons. Significances are referred to the negative control (CTRL–). ** *p* < 0.0021; **** *p* < 0.0001, ns (not significant).

The antiviral activity of the M15RL mixture produced by *P. gessardii* M15 was first investigated against HSV-1 and HCoV-229E in both co-treatment and virus pre-treatment experiments through the quantification of plaques reduction. HSV-1 and 229E were chosen to represent a viral model for enveloped DNA and RNA viruses, respectively [45,46]. In the co-treatment experiment, Vero cells were treated simultaneously with viruses and M15RL at different concentrations. In contrast, in the virus pre-treatment assay, the viral particles were firstly incubated with M15RL and then titrated on the cells. M15RL exhibited a strong inhibitory effect against HSV-1 in both experiments, with no significant difference between the two different assays showing complete viral inactivation at 6 µg/mL (Figure 1). As concern HCoV-229E, the inhibitory effect was significantly higher in the virus pre-treatment assay, showing 80% and 60% of inhibition at 6 and 3 µg/mL, respectively, as reported by Figure 1.

**Figure 2:**
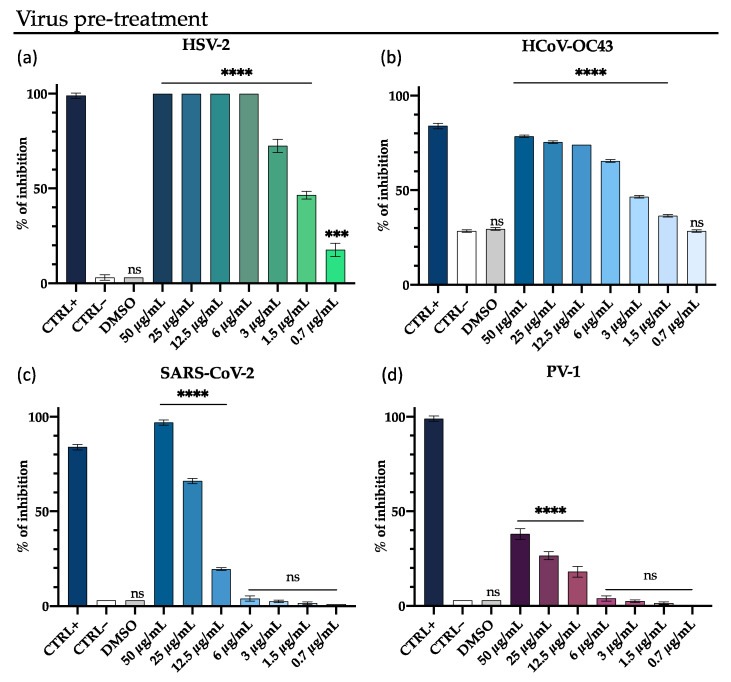
Antiviral activity of M15RL against (**a**) HSV-2, (**b**) HCoV-OC43, (**c**) SARS-CoV-2, and (**d**) poliovirus PV-1 in the virus pre-treatment assay (virus treated with M15RL for 1h and then added on cells). The Greco extract [42] at 50 µg/mL was used as a positive control (CTRL+). Data are means of three independent experiments. Statistical analyses were determined by ANOVA with Dunnett’s test for multiple comparisons. Significances are referred to the negative control (CTRL–). *** *p* < 0.0002, **** *p* < 0.0001, ns (not significant).

Due to better results obtained in the co-treatment assay against HCoV-229E, we decided to enlarge the viruses panel and test the M15RL mixture against other viruses belonging to *Coronaviridae* and *Herpesviridae* families with this modality. In particular, the antiviral activity of M15RL was investigated against the enveloped HSV-2, HCoV-OC43 and SARS-CoV-2, and non-enveloped poliovirus PV-1. M15RL showed an antiviral effect against HSV-2 with complete inhibition at 6 µg/mL and 70% at 3 µg/mL, similarly to HSV-1. As regards HCoV-OC43, 80% inhibition of the cytopathic effect is observed in the presence of M15RL at 25 µg/mL, and 50% at 3 µg/mL. In contrast, in the absence of M15RL, about 25% inhibition of the cytopathic effect is induced by the virus on the Vero cell monolayer. Furthermore, the mixture showed high activity also against SARS-CoV-2, with a complete viral inactivation at 50 µg/mL and 70% inhibition at 25 µg/mL. When the antiviral activity was also tested against the non-enveloped poliovirus PV-1, we observed a viral load reduction of less than 40% at 50 µg/mL (Figure 2).

**Figure 3:**
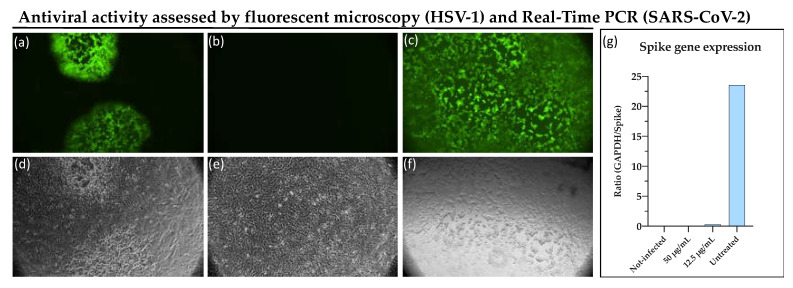
Antiviral activity of M15RL against GFP-HSV-1. Plaques can be visualized in fluorescent (**a**) and RGB microscopy (**d**) in cells treated with 3 µg/mL. No plaques are present in (**b**) and (**e**) where cells have been treated with 6 µg/mL of M15RL, while (**c**) and (**f**) show untreated infected cells in fluorescence and RGB microscopy, respectively. (**g**) Quantification of mRNA levels of the spike proteins expressed in Vero cells infected by SARS-CoV-2. Data are presented as ratio between the reference (GAPDH) and target (S) genes. Statistical analyses were determined by ANOVA with Dunnett’s test for multiple comparisons.

An additional test was also carried out to verify through fluorescence microscopy the direct action of M15RL on viral particles of HSV-1 (virus pre-treatment). HSV-1-GFP contains the Green Fluorescence Protein (GFP) in a viral genome sequence that encodes for tegument protein VP22 and makes infected cells fluorescent in green. From the captured images (Figure 3a–f), it is evident that, at the concentration of 6 µg/mL no fluorescence signal was detected, indicating that the virus was not able to enter into the cell in the presence of M15RL. In contrast, a partial cytopathic effect is observed at 3 µg/mL, reflecting an intermediate fluorescence compared to the negative control represented by the virus only. Fluorescence microscopy data confirmed results from the plaque reduction assay.

M15RL antiviral activity on SARS-CoV-2 was also evaluated by quantifying the relative expression levels of the mRNA coding for the Spike (S) protein, a transmembrane protein essential to penetrate the host cell and start the infection [47]. This feature is peculiar to the *Coronaviridae* family, giving its members the shape of a solar crown (corona) when observed under electron microscopy. No detectable levels of the genes coding for the S protein were recorded at the highest tested concentration (50 µg/mL), while reduced expression levels in comparison with the untreated sample were found at a lower concentration (12.5 µg/mL).

### 3.2. M15RL Mode of Action on HSV-1

The strong activity of M15RL against enveloped viruses, in contrast with the weak activity against the non-enveloped virus PV-1, could suggest a mode of action directed to the viral membrane. To further investigate in which step of the viral life cycle M15RL acts, we adopted several strategies, using HSV-1 as a model:

- Cells pre-treatment assay: the Vero cells were treated with M15RL and incubated for 1 h at 37 °C to investigate if action is also directed on the cells, preventing the viral infection. Then, cell monolayers were infected with HSV-1 for 1 h.
- Attachment assay: in most enveloped viruses, including HSV-1, viral entry/fusion is stopped at 4 °C [44,48]. Thus, cells were pre-chilled at 4 °C for 30 min, treated simultaneously with virus and M15RL, and incubated at 4 °C for 1 h, which allow viral attachment, but not penetration. This assay could evaluate if M15RL interferes with the virus binding to the cells.
- Fusion assay: cells were pre-cooled at 4 °C for 1 h, then infected and incubated at 4 °C for 1 h, allowing the virus to attach to the cells. Then the HSV-1 inoculum was removed, the cells treated with M15RL, and incubated for 1h to verify if the mixture could block the penetration of the virus in the host cell after its binding to the membrane.
- Post-treatment assay: the cells were first incubated with HSV-1 for 1 h at 37 °C, then M15RL was added and incubated for 1 h at 37 °C to study if the mixture could act on viral replication stages.
- Quantification of early and late viral genes: mRNA early gene UL52 levels, encoding for DNA primase, and late gene UL27, for structural envelope glycoprotein B, were quantified after 48 h of infection.

**Figure 4:**
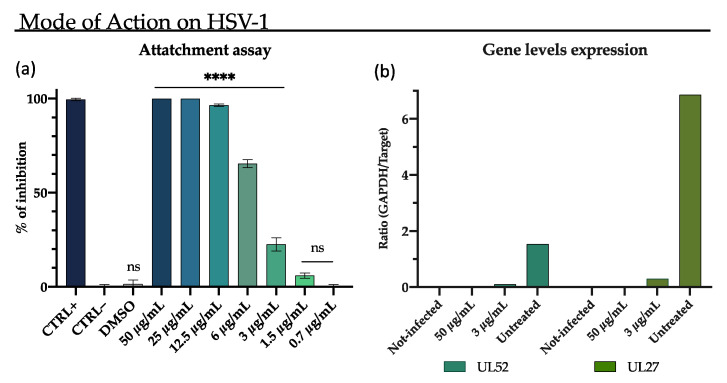
The M15RL’s mode of action on HSV-1 was investigated through (**a**) attachment assay and (**b**) expression levels of UL52, an early gene coding for DNA primase, and UL27, a late gene coding for glycoprotein B, respectively. Data are means of three independent experiments. Statistical analyses were determined by ANOVA with Dunnett’s test for multiple comparisons. Significances are referred to the negative control (CTRL–). **** *p* < 0.0001, ns (not significant).

No significative inhibition was observed at the tested concentration in the cell-pre-treatment, post-treatment, and fusion assay (Figure S3). Differently, M15RL was able to completely block the attachment of HSV-1 to the cells at 12.5 µg/mL and reduce it to the 60% at 6 µg/mL (Figure 4a). To further investigate in which stage of HSV-1 life cycle M15RL acts, the levels of early gene UL52 and late gene UL27 were quantized after treatment with M15RL. As shown in Figure 4b, expression levels of both genes were not detectable in the cells treated with 50 µg/mL of M15RL. This result confirms again that the mixture acts in the early stage of the infection.

These findings, along with that of the virus pre-treatment results, are compatible with a mode of action directed against the envelope of the viruses that led to the disruption of this structure and the subsequent inability of the viruses to attach themselves to the host cell, starting the infection.

#### Treated Virions Visualization

To visualize the effect of the mixture on the viral envelope, HSV-1 and SARS-CoV-2 virions were treated with M15RL and observed under transmission electron microscope (TEM). Again, outcomes confirmed the hypothesis that M15RL acts on enveloped viruses disrupting the envelope.

**Figure 5:**
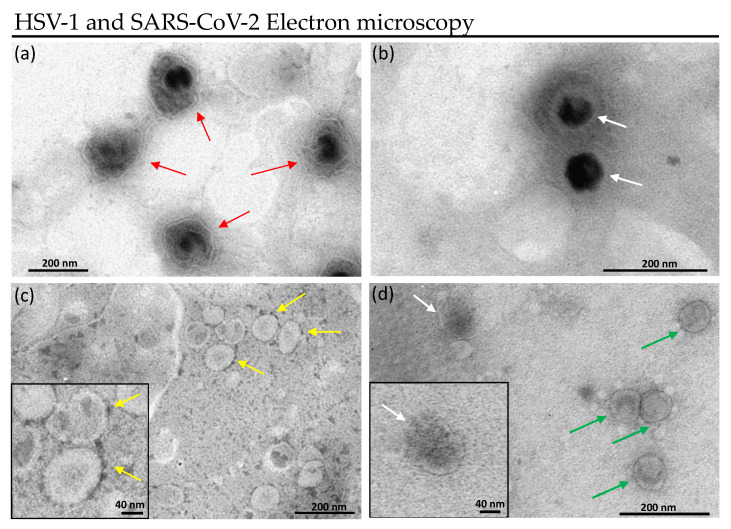
Transmission electron microscopy images of HSV-1 (**a**) and (**b**), and SARS-CoV-2 (**c**) and (**d**) virions. In (**a**), red arrows show untreated HSV-1 viral particles surrounded by the envelope (white ring) and tegument. The tegument represents the amorphous space between the nucleocapsid (black) and the envelope. In (**c**), spike proteins of untreated SARS-CoV-2 virions are indicated by yellow arrows. In (**b**) and (**d**), white arrows indicate naked viral particles of HSV-1 and SARS-CoV-2, respectively, treated with 50 µg/mL of M15RL. Green arrows indicate SARS-CoV-2 particles without S proteins. The black squares in (**c**) and (**d**) represent a magnification of a virion in the respective conditions.

HSV-1 particles are structured in core, nucleocapsid, tegument, and envelope. The tegument is unique to herpesviruses and consists of more than 15 proteins residing between the capsid and envelope, appearing amorphous in electron microscopy [49,50]. Figure 5a shows untreated HSV-1 in which red arrows indicate the viral particles surrounded by tegument and envelope. When treated with M15RL, viral particles lose both tegument and envelope, thus the ability to infect cells (Figure 5b).

SARS-CoV-2 virions consist of a nucleocapsid enclosed by the envelope, which presents the peculiar protruding S proteins that are anchored to the envelope [51]. Figure 5c shows SARS-CoV-2 virions with S proteins (indicated by yellow arrows). TEM results suggest that M15RL acts detaching S proteins from the envelope that led to the inactivation of the virus.

### 3.3. Antiviral Activity on RLs-Coated Surface

Eradicating the virus that remains active on the surfaces is crucial for preventing the diffusion of viral infections through fomites. To this aim, the antiviral activity of M15RL against HSV-1 and HCoV-229E on treated surfaces was investigated. Briefly, viruses were put in touch with wells coated with M15RL for 5 min, then titrated on Vero cells monolayer, and the activity was assessed by plaque reduction assay.

**Figure 6:**
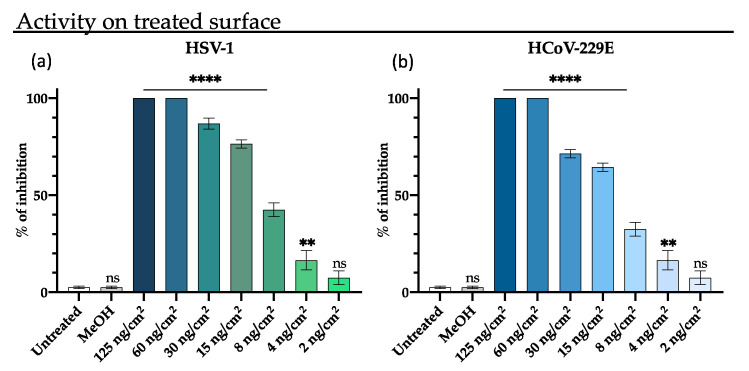
Virus inactivation on surfaces treated with M15RL. Wells bottom surfaces of a 12-well plate were coated with different quantities of M15RL. Afterward, 10^4^ PFU of HSV-1 (**a**) and HCoV-229E (**b**) were put in touch with the treated surfaces for 5 min, then the contact was interrupted, and the viruses were titrated on cells to test the virulence through plaque reduction assay. Data are means of three independent experiments. Statistical analyses were determined by ANOVA with Dunnett’s test for multiple comparisons. Significances are referred to the negative control (Untreated). ** *p* < 0.0002, **** *p* < 0.0001, ns (not significant).

M15RL coated surfaces exhibited very promising results, totally inactivating both viruses when treated with 66 ng/cm^2^ and reducing the viral load of 80% and 60% with 16 ng/cm^2^ for HSV-1 and HCoV-229E, respectively (Figure 6). As control, viral inhibition was not detected either when the plastic surface was treated only with the vehicle solvent (MeOH) or when viruses were put in touch with untreated plastic.

### 3.4. Rhamnolipids Cytotoxicity

The cytotoxic effect of M15RL was evaluated in vitro on two different cell lines, Vero and HaCaT, with the MTT assay. HaCaT are human keratinocyte cells spontaneously immortalized, widely used a model for epidermal cells [52,53]. For this reason, HaCaT was selected and used as epithelial cell model to evaluate the harmlessness of M15RL as potential detergents and disinfectants for the skin. 

**Figure 7:**
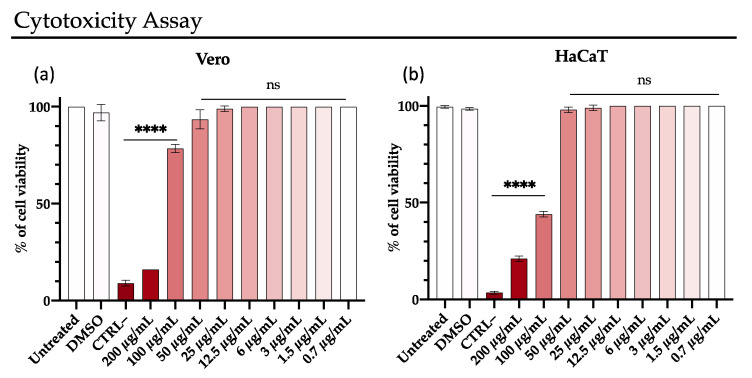
Cell viability evaluation by MTT assay on (**a**) Vero and (**b**) HaCaT cells after treatment with rhamnolipids for 24 h. 100 µL of DMSO was used as negative control (CTRL–). Data are means of three independent experiments. Statistical analyses were determined by one-way ANOVA with Dunnett’s test for multiple comparisons. Significances are referred to the untreated cells. **** *p* < 0.0001, ns (not significant).

The test reveals metabolically active cells, which can convert formazan to tetrazolium salts in the mitochondria. A significant reduction of the vitality was recorded at 200 µg/mL, while at 100 µg/mL, Vero and HaCaT cells showed 80% and 45% of viability, respectively. The RLs showed no toxicity for both cell lines used at the other tested concentrations (Figure 7).

## 4. Discussion

Viruses are divided into enveloped or naked, according to the presence or absence of the envelope, respectively [54]. Enveloped viruses represent a serious menace for global health and the economy. In fact, many diseases, pandemics, and thousands of deaths, such as Ebola virus disease, AIDS, influenza A, herpes, SARS, MERS, and COVID-19, are caused by enveloped viruses [55,56,57,58]. As recently experienced with COVID-19, outbreaks prevention and management require integrated approaches in combination with therapeutics [59]. In this context, the use of broad-spectrum antiviral agents, such as surfactants or alcohol-based sanitizers, represents one of the best strategies to prevent the spread of enveloped virus infections [60,61].

In this work, we demonstrated the antiviral activity of a mixture of 17 mono-rhamnolipids (M15RL), previously characterized by LC-MS/MS produced by the Antarctic marine strain *P. gessardii* M15 [40], against HSV-1, HSV-2, HCoV-229E, HCoV-OC43, and SARS-CoV-2. We also investigated the antiviral mode of action of the mixture towards HSV-1 and the ability to inactivate HSV-1 and HCoV-229E on coated surfaces.

M15RL was able to inhibit HSV-1 in co-treatment and virus pre-treatment assays without significant differences, while HCoV-229E was more efficiently inactivated by the pre-treatment assay. In fact, the mixture totally inactivated both HSV-1 and HSV-2 at the concentration of 6 µg/mL and showed 50% of inactivation at 1.5 µg/mL.

M15RL was also demonstrated to inactivate coronaviruses belonging to *alpha* and *beta* genera. Towards HCoV-229E showed complete plaque inhibition at 25 µg/mL and more than 60% of reduction at 3 µg/mL. Promising results were also achieved against SARS-CoV-2 that was totally inactivated at 50 µg/mL. Furthermore, the antiviral activity against SARS-CoV-2 was also proven, quantifying the expression levels of the mRNA coding for S protein. Moreover, even if the effects of biosurfactants on human coronaviruses (HCoV-229E and HCoV-OC43) have already been demonstrated, herein for the first time we report the antiviral activity of biosurfactants against the pandemic SARS-CoV-2 [62,63,64].

Few studies on the antiviral activity of biosurfactants against Herpes simplex viruses and Coronaviruses have been conducted so far. Recently, Jin et al. investigated the antiviral activity of commercial RLs 222B against HSV-1 and bovine coronavirus (BCoV), showing that 222B can inactivate both viruses at 90 µg/mL [38]. The activity of RLs against HSV-1 was also proven before by Remichkova et al. They showed that the mixture of two RLs, composed by one mono- and one di-rhamnolipid, can inhibit the cytopathic effect of HSV-1 and HSV-2 with IC_50_ of 14.5 µg/mL and 13 µg/mL, respectively. However, as the literature on the antiviral activity of RLs towards herpesviruses is very limited, we can only speculate that the higher activity of M15RL is due to its peculiar presence of 17 different RLs and in the uncommon high percentage of unsaturated RLs (60% of the mixture).

However, only comparable results against HSV-1 were shown by Palma Esposito et al., which identified novel trehalose lipids produced by a marine *Rhodococcus*, able to inactivate HSV-1 at 8 µg/mL [62]. Differently, these trehalose lipids showed lower antiviral efficacy than M15RL against HcoV229E. 

Although several works addressed the biological activity of biosurfactants for their ability to interfere with microbial or viral membranes [22,25,62,63], their mechanism of action is still not completely cleared. In this work, a panel of experiments to elucidate the M15RL mode of action were performed on the different HSV-1 life cycle steps.

The viral life cycle can be divided into three stages: entry, genome replication, and exit [65]. Entry is the first step of virus infection, and it starts with the attachment followed by the fusion with the host membrane, in the case of enveloped viruses [66]. The attachment mainly involves viral receptors on the cell membrane and viral antireceptors on the virus [65]. In enveloped viruses, the fusion of viral and cell membranes may proceed at the cell surface or after the internalization through an endosomal pathway of the virus particle [46]. Entry corresponds to the early viral life stage, while replication of the genome and exit from the cell represents the late phases of the infections.

Due to the possibility that M15RL may act either on the viral receptor or cellular antireceptors, cell pre-treatment and attachment assays were conducted [67].

Results showed that the M15RL can prevent the attachment of the viral particles to the cells, but this is not related to an action on the cells since cell pre-treatment has no effect. On the other hand, M15RL is unable to interfere with the fusion once the virus is attached to the cell membrane. Furthermore, the ability to counteract the infection when the virus penetrated the cell was evaluated by post-treatment assay, with negative results. Finally, RT-qPCR was utilized to quantify relative gene expression of early gene UL52, coding for DNA primase, and late gene UL27, coding for structural envelope glycoprotein B [68]. Once again, results show that expression levels of both genes in infected cells with pre-treated HSV-1 are significantly different from the levels expressed in infected cells at all. This finding confirms our hypothesis that M15RL inhibits the infection of enveloped viruses because blocking the binding of viral particles to cell membranes prevents the entry of the virus and consequently the replication of its genome.

Since viral receptors are anchored to the viral membrane and M15RL acts by inactivating selectively enveloped viruses, the antiviral activity could be related to an action directed on the envelope. To demonstrate our hypothesis and visualize the effect on the viral membranes, HSV-1 and SARS-CoV-2 were treated with M15RL and examined under the TEM, showing a disruption in the envelope integrity. This is in accordance with the literature’s evidence on RLs against HSV-1 [37,38]. Furthermore, M15RL low antiviral activity on naked poliovirus PV-1 suggests once again an action directed mainly towards the viral lipid envelope, supporting our hypothesis. Nevertheless, the electron microscopy images of RLs on SARS-CoV-2 are reported here for the first time.

The effectiveness of commercial RLs formulations, for example, as an additive to mask fabrics were recently reported against bovine coronaviruses [38]. 

Here, we show the ability of M15RL to eliminate active viral particles from contact surfaces. The results showed that M15RL was able to completely inactivate both members of herpesvirus and human coronavirus from treated surfaces when utilized at 60 ng/cm^2^.

Finally, we assessed the cytotoxicity of M15RL on two different cell lines—Vero, which we used for all the antiviral experiments and HaCaT cells that are widely reported as a model for human skin irritancy in vitro [53,69,70,71]. M15RL showed no toxicity at the active concentrations on both tested lines. Even if more studies are necessary, this finding, along with the antiviral activity, allows us to imagine the use of M15RL not only as a disinfecting agent for surfaces, but also as an additive in the formulation of topical medication.

## 5. Conclusions

Biosurfactants represent one of the most promising compounds in the pharmaceutical industry because of their structural versatility, stability and low toxicity that are useful in the design of therapeutics. Our experiments demonstrated the high antiviral activity of M15RL produced by the Antarctic bacterium *P. gessardii* M15 against viruses belonging to the *Coronaviridae* and *Herpesviridae* families, encouraging the application of these biosurfactants to counteract the widespread of viral pathogens. 

Considering all the outcomes, M15RL showed strong antiviral properties with a mode of action that could not lead to the development of resistance.

These findings suggest the opportunity to use the mixture M15RL produced by *P. gessardii* M15 as antiviral additives in formulations of hand sanitizers, lipsticks, intimate detergents, surface and household cleaners, but also in functionalizing surfaces and materials.

The market of surfactants is expected to grow in the coming years and, therefore, future research should be directed to develop eco-friendly ingredients for human and environmental safety. 

## Figures and Tables

**Table 1:** Main characteristics of the viruses utilized in this work.

Virus	Family	Genus	Nucleic Acid	Symmetry	Envelope	Dimensions
HSV-1	*Herpesviridae*	Simplexvirus	dsDNA	icosahedral	yes	155–240 nm
HSV-2	*Herpesviridae*	Simplexvirus	dsDNA	icosahedral	yes	155–240 nm
HCoV-229E	*Coronaviridae*	Alphacoronavirus	ssRNA(+)	helical	yes	80–120 nm
HCoV-OC43	*Coronaviridae*	Betacoronavirus	ssRNA(+)	helical	yes	80–120 nm
SARS-CoV-2	*Coronaviridae*	Betacoronavirus	ssRNA(+)	helical	yes	≈100 nm
PV-1	*Picornaviridae*	Enterovirus	ssRNA(+)	icosahedral	no	≈30 nm

ds: double-strand; ss: single-strand; (+): positive-sense strand.

**Table 2:** Primers used in the RT-PCR.

Host	Gene	Forward Sequence	Reverse Sequence
HSV-1	UL52	GACCGACGGGTGCGTTATT	GAAGGAGTCGCCATTTAGCC
HSV-1	UL27	GCCTTCTTCGCCTTTCGC	CGCTCGTGCCCTTCTTCTT
SARS-CoV-2	S	AGGTTGATCACAGGCAGACT	GCTGACTGAGGGAAGGAC
Vero	GAPDH	CCTTTCATTGAGCTCCAT	CGTACATGGGAGCGTC

## Data Availability

Not applicable.

## Supplementary Materials

The following are available online at https://www.mdpi.com/article/10.3390/pharmaceutics13122121/s1, Figure S1: Co-treatment on HSV-1 and HCoV-229E, Figure S2: Virus pre-treatment on HSV-1 and HCoV-229E, Figure S3: Mode of action on HSV-1. Table S1: Identified RLs present in M15RL.
