# Supplementary material for: Antiviral Activity of the Rhamnolipids Mixture from the Antarctic Bacterium Pseudomonas gessardii M15 against Herpes Simplex Viruses and Coronaviruses
Source: Pharmaceutics. 2021 Dec 8;13(12):2121. doi: 10.3390/pharmaceutics13122121 (PMC8704987; doi:10.3390/pharmaceutics13122121)
Supplement: Supplementary file 1 [file pharmaceutics-13-02121-s001.zip › pharmaceutics-1462572-supple.pdf]

pharmaceutics

MDPI

Supplementary Materials: Antiviral Activity of the Rhamnolipids Mixture from the Antarctic Bacterium *Pseudomonas gessardii* M15 against Herpes Simplex Viruses and Coronaviruses

Rosa Giugliano, Carmine Buonocore, Carla Zannella, Annalisa Chianese, Fortunato Palma Esposito, Pietro Tedesco, Anna De Filippis, Massimiliano Galdiero, Gianluigi Franci and Donatella de Pascale

Table S1. Identified RLs present in M15RL [a].

No.	Molecular Formula	RL	Relative Abundance %
1	$C_{24}H_{44}O_9$	Rha-C_8-C_{10}	5
2	$C_{25}H_{46}O_9$	Rha-C_9-C_{10}	<0.5
3	$C_{26}H_{46}O_9$	Rha-$C_{12:1}$-C_8	5
4	$C_{26}H_{48}O_9$	Rha-C_{10}-C_{10}	10
5	$C_{27}H_{50}O_9$	Rha-C_{11}-C_{10}	<1
6	$C_{28}H_{50}O_9$	Rha-$C_{12:1}$-C_{10}	19
7	$C_{28}H_{52}O_9$	Rha-C_{12}-C_{10}	14
8	$C_{30}H_{52}O_9$	Rha-$C_{12:1}$-$C_{12:1}$	8
9	$C_{30}H_{54}O_9$	Rha-$C_{14:1}$-C_{10}	13
10	$C_{30}H_{54}O_9$	Rha-C_{12}-$C_{12:1}$	2
11	$C_{30}H_{54}O_9$	Rha-$C_{12:1}$-C_{12}	5
12	$C_{32}H_{56}O_9$	Rha-$C_{14:1}$-$C_{12:1}$	<0.5
13	$C_{30}H_{56}O_9$	Rha-C_{14}-C_{10}	12
14	$C_{32}H_{58}O_9$	Rha-$C_{16:1}$-C_{10}	5
15	$C_{32}H_{58}O_9$	Rha-$C_{14:1}$-C_{12}	1
		Rha-C_{12}-$C_{14:1}$	
16	$C_{32}H_{60}O_9$	Rha-C_{16}-C_{10}	1

[a] Rha denotes the rhamnose moiety; $C_{x:y}$ means a fatty acid chain with X carbons and Y unsaturated bonds.

Co-treatment on HSV-1 and HCoV-229E

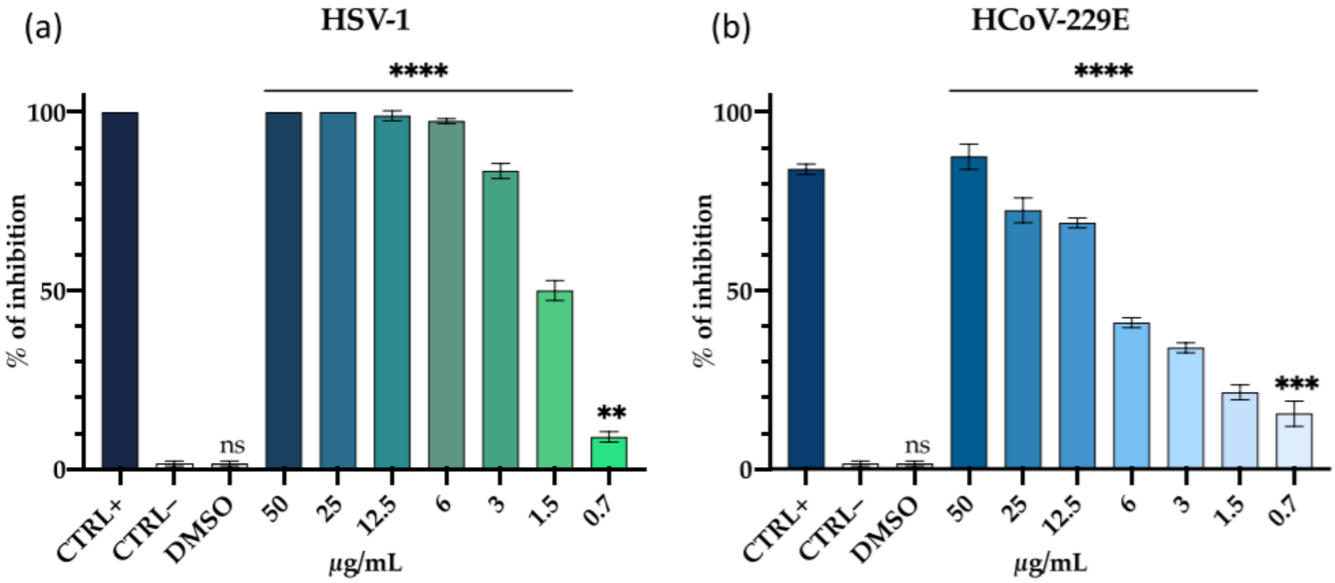

Figure S1. Antiviral activity of M15RL, presented as percentage of inhibition, against (**a**) HSV-1 and (**b**) HCoV229E in the co-treatment assay (cells treated with virus and M15RL at the same time). The inhibition percentage was calculated by comparing the number of plaques found in the presence of M15RL with respect to the untreated virus (CTRL–) (Formula 2). The Greco extract at 50 µg/mL was used as a positive control (CTRL+). Data are means of three independent experiments. Statistical analyses were determined by one-way ANOVA with Dunnett's test for multiple comparisons. Significances are referred to the negative control (CTRL–). ** $p < 0.0021$; *** $p < 0.0002$, **** $p < 0.0001$, ns (not significant).

Virus Pre-treatment on HSV-1 and HCoV-229E

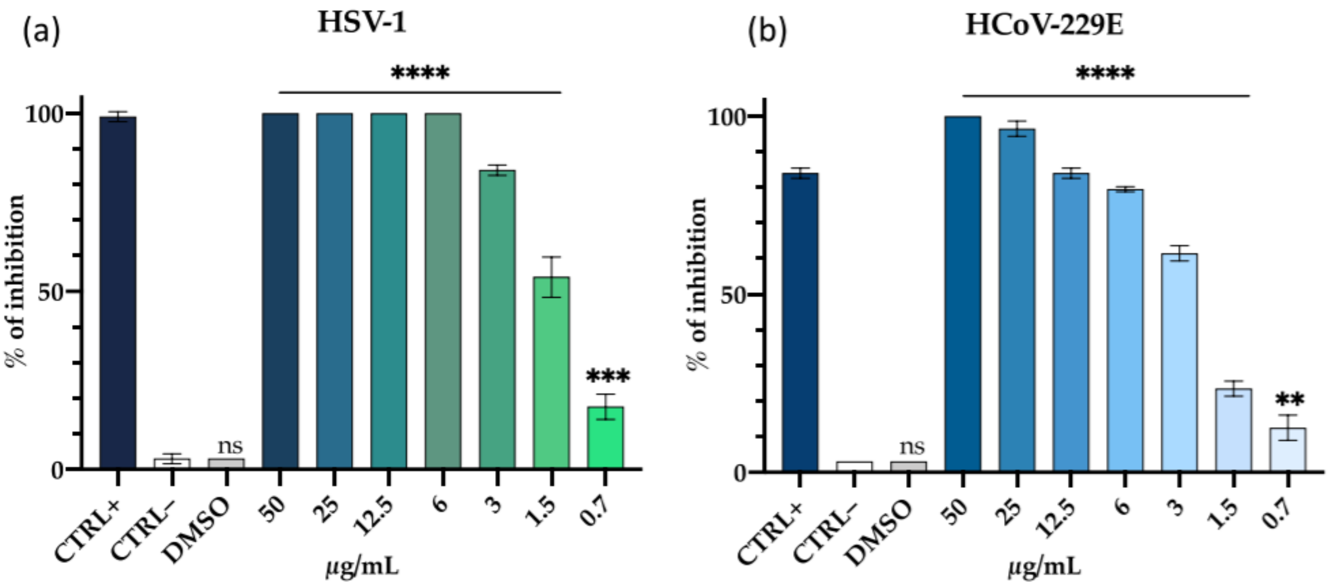

Figure S2. Antiviral activity of M15RL, presented as percentage of inhibition, against (**a**) HSV-1 and (**b**) HCoV229E in the virus pre-treatment assay (virus treated with M15RL for 1 h and then titrated on cells). The inhibition percentage was calculated by comparing the number of plaques found in the presence of M15RL with respect to the untreated virus (CTRL–) (Formula 2). The Greco extract at 50 µg/mL was used as a positive control (CTRL+). Data are means of three independent experiments. Statistical analyses were determined by one-way ANOVA with Dunnett's test for multiple comparisons. Significances are referred to the negative control (CTRL–). ** $p < 0.0021$; *** $p < 0.0002$, **** $p < 0.0001$, ns (not significant).

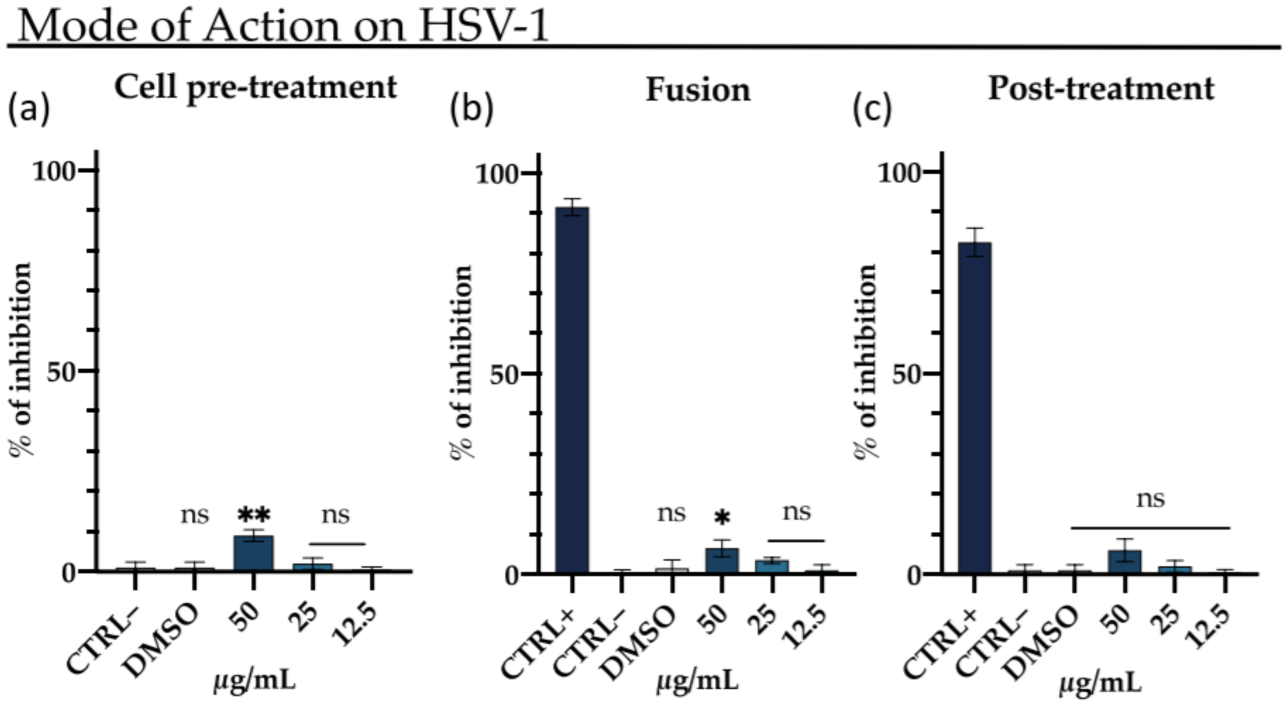

Figure S3. The M15RL's mode of action on HSV-1 was investigated through (**a**) Cell pre-treatment assay; (**b**) Fusion assay; and (**c**) Post-treatment assay. The inhibition percentage was calculated by comparing the number of plaques found in the presence of M15RL with respect to the untreated virus (CTRL–) (Formula 2). Data are means of three independent experiments. Statistical analyses were determined by ANOVA with Dunnett's test for multiple comparisons. Significances are referred to the negative control (CTRL–). * $p < 0.0332$; ** $p < 0.0021$, ns (not significant).
